# Effects of Metatarsal Foot Orthosis on Biomechanical 3D Ground Reaction Force in Individuals with Morton Foot Syndrome during Gait: A Cross-Sectional Study

**DOI:** 10.3390/life14030388

**Published:** 2024-03-14

**Authors:** Yongwook Kim

**Affiliations:** Department of Physical Therapy, College of Medical Sciences, Jeonju University, 303 Cheonjam-ro, Wansan-gu, Jeonju 55069, Republic of Korea; ptkim@jj.ac.kr

**Keywords:** ground reaction force, metatarsal orthosis, Morton foot syndrome

## Abstract

Morton’s foot syndrome (MFS) is characterized by a distally longer head of the second metatarsal bone compared to the head of the first metatarsal bone. Few studies have investigated the effects of a foot orthosis on kinetic characteristics, such as ground reaction force (GRF), during walking in individuals with MFS. This study aimed to verify dynamic GRF using a 3D motion analysis system, including two platforms with and without a foot orthosis condition. Kinetic GRF data of 26 participants with MFS were collected using a motion analysis system and a force platform. Participants were asked to walk wearing standard shoes or shoes with a pad-type foot orthosis. Repeated-measures analysis of variance (ANOVA) was used to compare the kinetic GRF data in the stance phase during gait according to the side of the leg and orthotic conditions for MFS. The late sagittal and frontal peak forces showed that the presence of a foot orthosis condition significantly increased the GRF when compared with the absence of a foot orthosis condition for both sides of the feet (*p* < 0.05). In addition, the second vertical peak force of the GRF showed that the presence of a foot orthosis condition significantly increased the GFR when compared with the absence of a foot orthosis condition on the side of the right foot (*p* = 0.023). Significant effects were observed in the late sagittal and frontal peak GRFs when wearing the pad-type foot orthosis in individuals with MFS during gait. Thus, even if there are no signs and symptoms of MFS in patients diagnosed with the disease condition, clinical interventions, such as a foot orthosis, that can be simply applied to shoe insoles are needed to manage and prevent various musculoskeletal disorders that may develop in the future. It was hypothesized that when wearing a foot orthosis, the participants would walk with increased GRF during gait compared to those without an orthosis.

## 1. Introduction

Morton’s foot syndrome (MFS) is characterized by a distally longer head of the second metatarsal bone compared to the head of the first metatarsal, hypermobility of the first metatarsal and first cuneiform, and hypertrophy and excessive weight load on the second metatarsal head [1,2,3]. It is known that about 20–25% of the population exhibits a short first metatarsal bone morphology, such as the Morton foot structure [4]. Although the primary etiology of MFS is not fully understood and remains controversial [5,6], it is a hereditary syndrome characterized by a short first metatarsal and posterior displacement of the sesamoid bones (sometimes leading to inflammation: sesamoiditis) [7]. Other theories have assumed replacement vascular or traumatic injuries as a cause of MFS [8].

The MFS is known to have a negative effect on biomechanical factors, such as pressure distribution and ground reaction force (GRF), in the Morton’s foot area during gait [3]. In addition, the syndrome usually causes severe pain and burning sensation in the intermetatarsal bones, which may spread to the adjacent toes, dorsum of the foot, and hindfoot [8]. MFS is exacerbated by walking using narrow shoes, and it is often associated with paresthesia or dysesthesia in the affected nerve region, known as neuroma [9,10]. However, most individuals with MFS perform daily activities, such as walking or jogging, without any specific clinical symptoms, such as metatarsalgia or paresthesia. Harris and Beath [11] evaluated the relative length of the first metatarsal using radiographs and self-developed footprints in 3619 men and performed a clinical examination of the foot. Approximately 32% of the participants reported that they had a shorter first metatarsal than the second metatarsal, and most of them did not have any clinical symptoms in the feet and toes [11]. Although the study by Harris has advantages due to its large sample size, the findings cannot be generalized to all individuals with MFS because most of the study subjects were young men and the symptoms of MFS appear later in life [5].

Sustained abnormal alignment and repetitive movement patterns of the first and second metatarsal structures are related to non-specific chronic metatarsalgia, pes planus, and hallux valgus deformity [3,12]. The characteristics of patients with MFS are imbalances in the muscle activity of the flexor hallucis brevis, abductor hallucis, and adductor hallucis during gait [13]. These muscles directly or indirectly affect the axis of the first metatarsal joint motion. In addition, a previous study reported that peak loading pressures and impulse values occurring under the second metatarsal head in the MFS group were significantly higher than those in the non-MFS group [2]. Therefore, even if there are no symptoms of MFS, early evaluation and management of individuals with the Morton foot structure are important to prevent future musculoskeletal disorders of the feet and toes and to reduce socioeconomic costs.

The clinical interventions for MFS include foot orthoses, intrinsic foot muscle exercise in physical therapy, medication, and surgical therapy [3,4,7]. Among these, foot-toe orthosis and shoe modifications can play an important role in the nonsurgical intervention of the metatarsophalangeal (MTP) pathology. A therapeutic foot-toe orthosis may improve the biomechanical gait efficiency; on the other hand, an inappropriate foot-toe orthosis can be a contributing factor for the development of the pathology and worsening of the symptoms [14]. Foot-toe orthoses should decrease the forefoot pain, reduce the abnormal weight pressure exerted on the lower extremity joints and foot segments, and increase the walking efficiency of subjects with MFS. However, previous studies on a foot-toe orthosis for MFS have verified the comfort of wearing an orthosis and the change in pain occurring beneath the first or second MTP joints using a simple measurement method, such as the visual analog scale (VAS) [4,15]. Objective and quantitative verification of the effects of orthotic intervention in MFS on the biomechanical GRF using a high-tech three-dimensional (3D) gait analysis system and a force platform is a very important issue in managing and preventing various musculoskeletal disorders. In addition, biomechanical analysis using a 3D motion analysis system is known globally as the best scientific verification method among methods for analyzing posture and special movements such as gait [16,17,18].

Therefore, the purpose of this study was to investigate the effect of pad-type foot-toe orthoses for supporting MFS on the GRF and moment of the ankle joints in individuals with MFS using a 3D motion analysis system during gait. It was hypothesized that when wearing a foot orthosis, the participants would walk with increased GRF during gait compared to those without an orthosis.

## 2. Materials and Methods

### 2.1. Participants

The participants were twenty-six adults (16 males and 10 females) with MFS. G*Power (version 3.1.9.7) analysis was used to estimate the appropriate sample size. A sample size of 20 was obtained based on an effect size large enough to identify the estimated significance based on a previous study [3] that examined the effects of intrinsic foot muscle exercise combined with interphalangeal flexion exercise on biomechanical variables. The inclusion criteria were an at least 8 mm more distally placed second metatarsal compared to the first metatarsal head in both feet and no severe pain in the metatarsal area that might hinder walking or a history of foot surgery. Potential participants were excluded if they had foot-toe osteoarthritis, neurological or musculoskeletal disorders of the foot and ankle joints, or if they were taking any medications that could interfere with walking. The participants self-reported the intensity of the foot and toe pain using a 10-cm VAS. Foot structures of the participants were evaluated for different positions of the metatarsal head and diagnosed with MFS by a licensed podiatric physician with 12 years of clinical experience in the local hospital. The evaluation was based on radiographic measurements according to the Harris method [11]. All participants fully understood the aim and procedure of the study, and they voluntarily participated in this study. The mean age, height, and weight of all participants were 33.2 ± 6.4 years, 168.9 ± 8.7 cm, and 62.6 ± 12.0 kg, respectively (Table 1). This research has been approved by the Jeonju University Institutional Review Board of the authors’ affiliated institutions (jjIRB-190611-HR-2019-0602). All participants provided written informed consent before study participation.

### 2.2. Experimental Materials and Biomechanical Analysis Instrumentation

**Figure 1:**
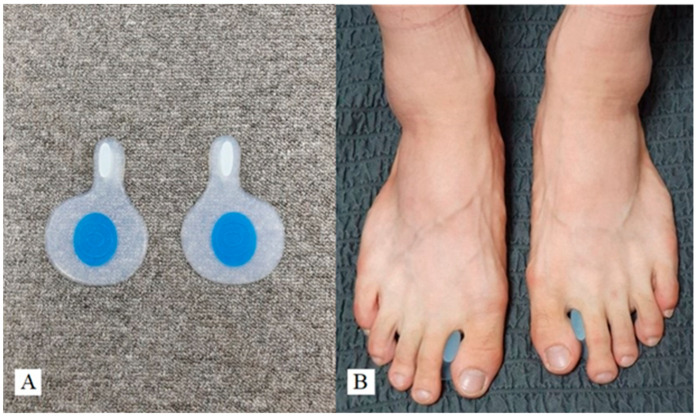
A soft silicone pad-type foot-toe orthosis (**A**) and application of a foot-toe orthosis (**B**).

The 3D GRF and moment data of the ankle joints were collected using a Vicon Motion Analysis System (Vicon Inc., Oxford, UK) with six cameras (T10) operating at a 100 Hz sampling rate and two BP400600 force platforms (AMTI, Watertown, MA, USA) synchronized with the camera system, which were embedded in the middle of the walkway and sampled data at 500 Hz. The captured kinematic and kinetic data were processed using the Nexus 1.8.5 software program (Vicon Inc., Oxford, UK). A calibration T-wand (7.5 cm) was used to calibrate the motion analysis system and to identify the lab origin. Nexus 1.8.5 software (Vicon Inc., Oxford, UK) was used to process the captured moment and GRF data in 3D space. The foot-toe orthosis (ZESPA Co., Seoul, Republic of Korea) for MFS allowed for the placement of a soft silicone pad-type between the hallux and the second toe (Figure 1).

### 2.3. Data Acquisition and Processing Procedure

**Figure 2:**
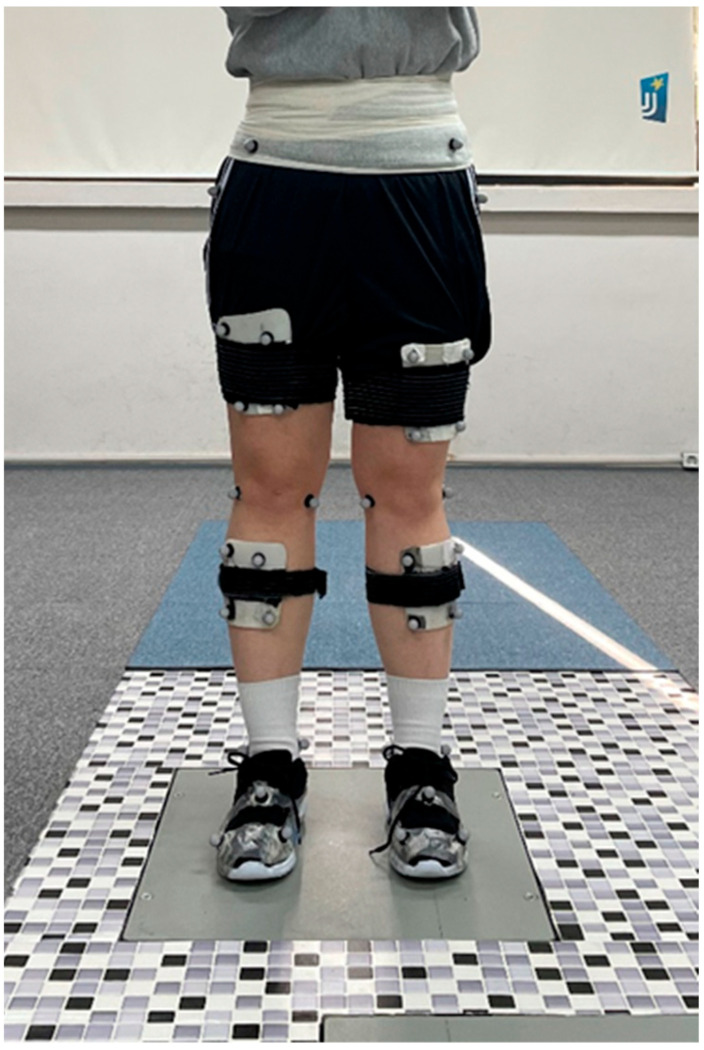
Forty infrared reflective markers placed on the participant’s pelvic and lower extremities to obtain the static calibration skeletal model and kinetic data.

A total of forty reflective markers (14 mm) were attached bilaterally to the participant’s anterior and posterior superior iliac spine, greater trochanter, femoral epicondyle, malleolus, rear foot, mid-foot, and fore-foot; and four cluster reflective markers were attached bilaterally to the thigh and lower leg segment according to the six-degrees-of-freedom (6DOF) model (Figure 2) [19]. Prior to obtaining the 3D biomechanical motion capture data of the lower extremities, static calibration data were collected from each participant to make a sample model for later analysis of the GRF during the gait trials. Following data acquisition of static calibration capture, the calibrated anatomical system technique was used to determine the changes in the kinetic GRF data of the lower extremities while the participants walked freely under two different foot-toe orthosis conditions, with and without a foot-toe orthosis (Figure 2). The participants were asked to walk wearing a standardized shoe provided by an experimenter along a 6 m walkway at their self-selected speed to obtain the 3D GRF data from two force platforms installed in the middle of the walkway in the laboratory. A total of 8 to 10 walk trials were performed for the with and without a foot-toe orthosis condition, and the average GRF data were analyzed bilaterally through all walk trials. The walking trials were performed in only one direction. Under the same orthosis conditions, the walking experiment was conducted without any pause between gait trials, except for unexpected situations such as the detachment of the reflective markers. The application order of the orthosis conditions was assigned randomly before the experimental trials were started.

**Figure 3:**
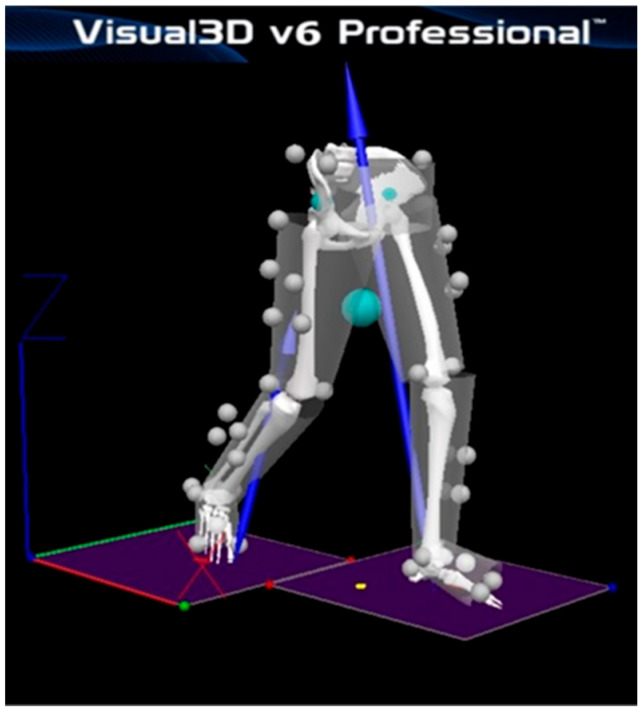
Visual3D representation of 40 infrared reflective markers and dynamic ground reaction peak force (blue arrow) in the stance phase during a gait trial.

Visual3D analysis software v6 professional (C-Motion Inc., Germantown, MD, USA) was used to analyze the final GRF data following data acquisition using the Vicon Nexus software program and the 3D motion capture system. Visual3D software created a virtual static skeletal model for each subject based on reflective marker settings. The virtual static skeletal model was used to produce all virtual walking models during experimental gait trials (Figure 3). The X-Y-Z Cardan sequence was used to define the order of the rotations following the right-hand rule for the segment coordinate system axes [19]. The 3D GRF data were low-pass filtered with a fourth-order Butterworth filter and a cutoff frequency of 15 Hz. The Visual3D analysis program produced a visual representation of the magnitudes and directions of the GRF bilaterally in space, which enabled the calculation of the related stance phase (Figure 3). The GRF data were normalized for body weight.

### 2.4. Statistical Analysis

The Kolmogorov–Smirnov test was used to confirm that the GRF data showed a normal distribution. Two-way repeated-measures ANOVA was used to verify the effects of with and without orthosis condition and foot side. When a significant F-value was confirmed, the Bonferroni post hoc test was used to determine the pairwise comparison. The 3D GRF and ankle moment data that occurred bilaterally during each stance phase point were used for comparison between the with and without orthosis conditions. All analyses were conducted using SPSS version 26.0 (IBM Corp., Armonk, NY, USA). Differences were considered significant at the α = 0.05 level.

## 3. Results

### 3.1. 3D GRF with vs. without a Foot Orthosis and Sides of the Feet in the Stance Phase during Walking

Table 2 shows the peak GRF that occurred in the 3D movement plane with and without a foot orthosis during gait. There were significant differences in the GRF variables between the orthosis conditions in various stance phases of the gait cycle (Table 2). The late peak force caused sagittal and frontal motion planes during loading (75–100% of the stance phase), and the second vertical peak force of the GRF showed significant differences according to the orthosis conditions (*p* < 0.05) (Table 2). However, there were no significant differences in any GRF variables between the sides of the feet (*p* > 0.05) (Table 2). Also, there were no interactive effects between the orthosis conditions and sides of the feet in any of the GRF values (*p* > 0.05) (Table 2).

### 3.2. Mean and Standard Deviation of the 3D Peak GRF with vs. without Foot Orthosis according to Sides of the Feet

The results of a 2 × 2 repeated-measures ANOVA comparing the 3D GRF that developed in the stance phase during gait with or without a foot orthosis according to the sides of the feet are shown in Table 3. The late sagittal peak force (75–100% of the stance phase) showed that the presence of a foot orthosis condition significantly increased the GRF when compared with the absence of a foot orthosis for the side of the right foot (*p* = 0.001) and the side of the left foot (*p* = 0.002) (Table 3). In addition, the late frontal peak force of the GRF showed that the presence of a foot orthosis condition significantly increased the GFR when compared with the absence of a foot orthosis condition for both sides of feet (*p* < 0.05) (Table 3). The second vertical peak force of the GRF showed that the presence of a foot orthosis condition significantly increased the GFR when compared with the absence of a foot orthosis condition on the side of the right foot (*p* = 0.023) (Table 2). However, there were no significant differences in the other GRF variables between the foot orthosis conditions and sides of feet (*p* > 0.05) (Table 3).

## 4. Discussion

The study investigated the characteristics of peak GRF variables according to the presence or absence of a foot orthosis condition using two force platforms and 3D motion analysis during normal gait in individuals with MFS. Reliable and objective biomechanical data acquisition is an essential process to confirm the characteristics of related musculoskeletal dysfunction, and it is an important component for decisions on benign therapeutic interventions in patients with foot-toe deformities, such as MFS. The results showed that the maximal GRFs were significantly increased in the late sagittal peak force (developed 75–100% stance phase) when applying a foot orthosis condition compared to the absence of a foot orthosis condition in both feet. Additionally, late frontal peak GRFs that occurred in both legs were significantly increased in the presence of a foot orthosis condition compared to the absence of a foot orthosis condition. The repulsive force developed on the ground during gait affects the biomechanical values of the joints and segments of the lower extremities and plays an important role in performing daily activities; thus, it is known as a major variable in the evaluation of gait patterns and balance function [20,21]. The results of this study showed increased GRF values in the late anteroposterior and mediolateral maximal forces in both feet and the second vertical maximal force on the side of the right leg when the foot orthosis was applied. Positive changes in the GRF variables according to the foot orthosis effect appeared in the final stage of the stance phase during walking. Although no previous studies have compared these results directly, this suggested that a foot orthosis applied to the first and second MTP joint areas beneath the inner side of the forefoot caused a change in the GRF by increasing the pushing force of the ground, which plays a biomechanically important role in the final stage of the stance phase during walking. In addition, these changes will cause vigorous early swing phase acceleration, which will have a positive effect on the quality and quantity of gait patterns. Results showed no significant changes in the GRF values in the early stance phase with and without metatarsal foot orthosis conditions during gait. However, in some participants, the orthotic gait condition increased the initial sagittal peak force, and the first vertical peak force developed a 0–50% stance phase. This indicates that the improvement of propulsion force through the application of the metatarsal foot orthosis on one foot during the double-stance period affected the initial GRF of the opposite foot.

The skeletal structures of the first MTP and metatarsocuneiform joints and the medial aspect of the capsulo-ligamentous distribution of the forefoot represent the main static stabilizers that allow stability during weight transfers in the stance phase [4]. The lateral ligaments between the first metatarsal head and the base of the first proximal phalanx offer static and dynamic stability to the first MTP joint [22]. Among the signs and symptoms of MFS, hypertrophy of the second metatarsal bone causes dysfunction, and metatarsalgia in the medial forefoot and inflammation of the metatarsal joint are common [5]. In general, metatarsalgia, including burning sensations and discomfort, occurs during gait or when it is followed by excessive exercise and prolonged standing. These musculoskeletal disorders are due to excessive GRF development in the second metatarsal joint area during push-off in the late stance phase [2,5]. For balance and stability of the entire body with an even distribution of weight, the three points of the foot (the first and fifth metatarsal heads and the calcaneus) must come into contact with the ground in a tripod-like manner. However, if the second metatarsal bone is long, there is development of lateral movement of the weight support point responsible for the first metatarsal bone and an imbalance in weight distribution and ankle instability. From the biomechanical aspect, this imbalance related to an abnormal gait pattern and poor postural alignment may cause musculoskeletal problems in the knee, hip, back, and neck, and even headaches [23,24]. Therefore, clinical interventions, such as foot braces, applied in this study are necessary for patients with MFS to maintain their overall musculoskeletal balance and to manage and prevent various musculoskeletal disorders that may occur in the future.

The current study examines the metatarsal foot orthosis for MFS to confirm their effects on the GRF using a force platform and a quantitative, high-technology 3D gait and motion analysis system. This study had several limitations. Although the study subjects had MFS, this study was conducted among individuals in relatively good health without pain in the feet and toes or any musculoskeletal problems. Therefore, it is difficult to generalize the results of this study to all MFS patients with musculoskeletal signs and symptoms. In addition, because this study was conducted using a cross-sectional study design, we did not verify the long-term effects of foot orthosis application for the management of MFS. Due to the difficulty of biomechanical analysis, we could not perform verification of various kinetic parameters that occur in various lower extremity joints. Further studies are necessary to verify the long-term effects on kinetic and kinematic changes according to various clinical intervention methods, including a foot orthosis, for patients with MFS.

## 5. Conclusions

In conclusion, the results showed significant effects in the late sagittal and frontal peak GRFs when wearing the pad-type foot orthosis in individuals with MFS during gait. Therefore, even if there are no signs and symptoms of MFS in patients diagnosed with the disease condition, the clinical intervention of a foot orthosis that can be simply applied to shoe insoles is needed to manage and prevent various musculoskeletal problems that may develop in the future.

## Figures and Tables

**Table 1:** Characteristics of the study participants (*n* = 26).

Characteristics	Mean ± SD
Gender (male/female)	16/10
Age (years)	33.2 ± 6.4
Height (cm)	168.9 ± 8.7
Weight (kg)	62.6 ± 12.0
Gait speed (m/s)	1.32 ± 0.40
Step length (m)	119.18 ± 8.85
Step width (m)	9.75 ± 2.12

SD: standard deviation.

**Table 2:** Repeated-measures ANOVA comparing 3D ground reaction force according to each orthotic condition and foot side (*n* = 26).

Peak Force Variables (N/kg)	Level	F	*p* Value
ISPF	Orthosis conditions	1.564	0.217
	Foot sides	0.521	0.467
	Conditions × sides	0.961	0.350
LSPF	Orthosis conditions	8.027	0.010 *
	Foot sides	1.520	0.226
	Conditions × sides	1.938	0.154
IFPF	Orthosis conditions	0.888	0.377
	Foot sides	1.443	0.256
	Conditions × sides	2.557	0.095
LFPF	Orthosis conditions	3.122	0.040 *
	Foot sides	1.140	0.291
	Conditions × sides	1.577	0.209
1st VPF	Orthosis conditions	1.828	0.192
	Foot sides	0.544	0.451
	Conditions × sides	0.967	0.339
MMF	Orthosis conditions	1.520	0.228
	Foot sides	0.268	0.740
	Conditions × sides	0.810	0.385
2nd VPF	Orthosis conditions	3.910	0.037 *
	Foot sides	1.137	0.294
	Conditions × sides	0.780	0.394

* *p* < 0.05. ISPF: initial sagittal peak force, LSPF: late sagittal peak force, IFPF: initial frontal peak force, LFPF: late frontal peak force, VPF: vertical peak force, MMF: midstance minimal force.

**Table 3:** Peak ground reaction force with and without foot orthosis during the stance phase of normal walking (*n* = 26).

Foot Sides	Variables (N/kg)	Orthosis	No Orthosis	*p*
Right foot	ISPF	1.92 ± 0.33	1.84 ± 0.37	0.274
	LSPF	2.20 ± 0.29	2.13 ± 0.19	0.001 *
	IFPF	0.61 ± 0.16	0.59 ± 0.15	0.614
	LFPF	0.60 ± 0.14	0.54 ± 0.11	0.003 *
	1st VPF	10.87 ± 1.20	10.69 ± 1.19	0.372
	MMF	6.88 ± 1.17	6.78 ± 1.22	0.441
	2nd VPF	11.25 ± 1.22	10.49 ± 0.94	0.023 *
Left foot	ISPF	1.89 ± 0.31	1.81 ± 0.31	0.110
	LSPF	2.18 ± 0.22	2.11 ± 0.25	0.002 *
	IFPF	0.59 ± 0.29	0.57 ± 0.31	0.711
	LFPF	0.58 ± 0.11	0.52 ± 0.15	0.014 *
	1st VPF	10.66 ± 1.21	10.54 ± 1.30	0.626
	MMF	7.06 ± 0.73	6.92 ± 0.85	0.351
	2nd VPF	11.11 ± 1.33	10.76 ± 1.07	0.057

Values are presented as mean ± SD. * *p* < 0.05, significant differences between with and without foot orthotic condition using 2 × 2 repeated measures ANOVA with Bonferroni’s correction. Peak sagittal, frontal, and vertical ground reaction force calculated in the stance phase during normal speed walking. ISPF: initial sagittal peak force, LSPF: late sagittal peak force, IFPF: initial frontal peak force, LFPF: late frontal peak force, VPF: vertical peak force, MMF: midstance minimal force.

## Data Availability

Not applicable.
